# Burnout Stigma Inventory: Initial Development and Validation in Industry and Academia

**DOI:** 10.3389/fpsyg.2020.00391

**Published:** 2020-03-12

**Authors:** Ross W. May, Julia M. Terman, Garett Foster, Gregory S. Seibert, Frank D. Fincham

**Affiliations:** ^1^Family Institute, The Florida State University, Tallahassee, FL, United States; ^2^Department of Psychological Science, University of Vermont, Burlington, VT, United States; ^3^Maritz, St. Louis, MO, United States; ^4^Department of Psychiatry, University of California, San Diego, San Diego, CA, United States

**Keywords:** burnout, instrument development, item response theory, mental health, stigma

## Abstract

Although burnout is a risk factor for various negative mental and physical outcomes, its prevention is hampered by the stigma associated with burnout. The current research therefore reports on the initial development and validation of a novel measure of perceived burnout stigma. Study 1 (*n* = 318) describes the construction and initial evaluation of scale items derived from established mental health stigma and burnout scales. Study 2 (*n* = 705) then replicated the burnout stigma factor structure established in the initial study. Additionally, it evaluates relationships between occupational and school burnout stigma and indicators of mental health. Hierarchical multiple regressions showed that burnout stigma accounted for variance in depression, anxiety, and stress over and beyond that of burnout. Study 3 (*n* = 682) extended these findings via cross-lagged and bidirectional models, demonstrating that burnout stigma predicted mental health indicators 6 weeks later. Study 4 (*n* = 717) supplemented earlier exploratory and confirmatory factor analyses using item response theory to further demonstrate that perceived burnout stigma is a unidimensional construct potentially applicable to both work and school settings. Overall, the current research resulted in an eight-item burnout stigma instrument (BSI-8) with excellent psychometric properties that predicts indicators of mental health.

## Introduction

Although conceptualizations vary, burnout is widely considered to comprise three factors (emotional exhaustion, cynicism toward one’s work, and doubt in professional efficacy) resulting from prolonged exposure to work-related stress (Maslach et al., 2001; Schaufeli et al., 2009; Maslach, 2017). Highlighting its growing importance in relation to wellness, the World Health Organization (WHO) recently announced that the upcoming 11th Revision of the International Classification of Diseases (ICD-11) will provide a more detailed description of burnout, emphasizing burnout as a syndrome resulting from “chronic workplace stress that has not been successfully managed” (World Health Organization, 2019). Initially identified in human service occupations (Freudenberger, 1974), research has since documented burnout in other occupations (Maslach et al., 2001), and in academic (school) populations (Walburg, 2014). The application of burnout to students suggests that both employment and school obligations entail achievement pressures and that burnout manifests from difficulties in coping with those pressures (for a detailed commentary see Schaufeli and Taris, 2005; Salmela-Aro et al., 2009; Parker and Salmela-Aro, 2011). Indeed, data suggests that burnout levels are comparable in occupational and school settings (Reis et al., 2015) with both lay and research audiences noting concern about its “epidemic” prevalence (Milken Institute Center for the Future of Aging, 2018; Ranjbar and Ricker, 2018).

The fact that burnout is linked to numerous negative outcomes emphasizes its importance. Psychologically, for example, individuals experiencing burnout display cognitive impairments (Deligkaris et al., 2014), difficulty with emotional response suppression (Golkar et al., 2014), and increased levels of clinical depression (Bianchi et al., 2013). In regards to physiology, burnout has been associated with differential brain structure (Savic, 2015), hypothalamic-pituitary-adrenal (HPA) axis dysregulation (Oosterholt et al., 2015), suboptimal cardiovascular functioning (May et al., 2014a, b, 2016, 2018) and increased risk of heart disease and mortality (Toker et al., 2012). In sum, burnout is a ubiquitous phenomenon of interest to a diverse audience including physiologists, mental and behavioral health professionals, and policy makers.

Even though ample evidence indicates that burnout is an independent risk factor for numerous deleterious mental and physical health outcomes, there are barriers to its prevention. One notable barrier is the stigma associated with burnout (Bianchi et al., 2016). The importance of stigma is emphasized in research on mental health where it has been shown to result in feeling insecure, inadequate, inferior, and weak; it also encourages avoidance, prejudice, and rejection of people with mental health conditions (Lannin et al., 2016). Thus, individuals with mental health challenges often face social consequences in addition to their health challenges. Some research suggests that stigma may even be more harmful to people with a mental health condition than the condition itself (Cechnicki et al., 2011).

The World Health Organization (WHO) considers stigma to be one of the greatest barriers to the treatment of mental health challenges (Orel, 2007). People presenting with symptoms are likely to perceive higher stigma than those without current symptoms (Busby Grant et al., 2016). This is problematic as stigma increases the risk of developing depression and anxiety (Pyle et al., 2015) and serves as a barrier for help-seeking behaviors (Bianchi et al., 2016). Several types of stigma affect those with mental health challenges, including perceived stigma. Perceived stigma refers to an individual’s beliefs about others’ attitudes toward mental health challenges (Busby Grant et al., 2016).

Applied to burnout, perceived stigma may reflect the belief that most people view burnt out individuals as less competent than those who are not burnt out. Perceived stigma is often internalized in the form of self-stigma, which in turn, predicts help-seeking attitudes and behaviors (Chronister et al., 2013; Jennings et al., 2015). Therefore, stigma is a powerful social force that has the potential to prevent treatment seeking and exacerbate the stigmatized challenges (Jennings et al., 2015; Pyle et al., 2015). This study explores burnout stigma conceptualized as the perceived stigma of individuals who experience burnout.

Like indicators of mental health, burnout carries stigma while also being socially contagious (see the overview provided in Bakker et al., 2005). As burnout manifests in both behavioral and social symptoms, symptoms can be noticed by others and incorporated into emotional contagion processes (i.e., mimicry). This may then result in a double-edged problem: burnout prevalence grows (as the likelihood of self-labeling increases), while the likelihood of help-seeking behaviors decreases (thus preventing appropriate treatment). Unfortunately, even though burnout stigmatization has attracted increasing interest from researchers in Europe (Bianchi et al., 2016, 2019), almost nothing is known about burnout stigma in the U. S in either employed or student populations. Establishing a thorough understanding of burnout stigma and its correlates is imperative in order to increase awareness and provide a platform for advocacy and policy change.

Therefore, to expand understanding of burnout stigma in the U.S. we investigated perceived burnout stigma in four studies using American samples. Study 1 first reports beliefs about the potential stigmatization of school burnout. Study 1 presents a new burnout stigma measure that can be adapted for use in academic and work settings. Study 2 confirms the factor structure identified in Study 1 and then documents mental health correlates in both academic and occupational samples. Study 3 then provides data on the temporal ordering of perceived burnout stigma and mental health indictors. Finally, Study 4 provides an item response theory analysis of the new eight-item burnout stigma instrument (BSI-8). This research emphasizes the importance of burnout stigma, describes the development and validation of a novel perceived burnout stigma measure that can be used in both occupational and academic settings, and highlights the potential influence of perceived burnout stigma on mental health indicators. Given recent attention aimed at increasing awareness of burnout symptomology, the development of a validated burnout stigma measure would provide an important contribution to burnout prevention and early identification efforts.

## Study 1

Burnout appears to be a serious problem worldwide (Sablik et al., 2013) that is linked to a variety of mental health challenges, including anxiety, depression, stress, and borderline personality traits (Mohammadi, 2006; Bianchi et al., 2013, 2018). However, research examining burnout stigmatization has only recently emerged (Dyrbye et al., 2015; Bianchi et al., 2016; Mullen and Crowe, 2017). Bianchi and colleagues were the first to study burnout-specific stigma (Bianchi et al., 2016). To measure burnout stigma, the authors replaced the term “depression” with “burnout” in a 7-item depression instrument derived by the authors (Crisp et al., 2005; Beck et al., 2009; Schwenk et al., 2010). Findings indicated that burnout was stigmatized at only a slightly lower level than depression (Bianchi et al., 2016), although other findings indicate that burnout may not be less stigmatized than depression (Mendel et al., 2015).

In any event, stigma related to burnout may be different from that related to depression (for further commentary contrasting burnout and depression, see Koutsimani et al., 2019). Burnout is less publicized than depression and is usually conceptualized within the specific context of work or school (Maslach et al., 2001; Schaufeli et al., 2009), whereas depression is viewed as context independent. Additionally, as each label (burnout and depression) potentially carries a unique social stigma, burnout and depression stigma may lead to differing outcomes. Thus, it may be worthwhile to examine burnout stigma as separate from depression stigma (Bianchi et al., 2016).

As burnout stigma in the U.S. has been underexplored, Study 1 sought to provide some content validation to the construct of burnout stigma. In order to better understand the stigma of a specific group, one must first ask those in the group for their thoughts about the group identity and stigma (Corrigan, 2018). Consequently, we first evaluated the awareness of burnout amongst students and their thoughts about burnout stigma. Although some research has studied stigma as it relates to burnout, this research has not explored student attitudes toward the construct of school burnout and burnout stigma (Dyrbye et al., 2015; Mullen and Crowe, 2017; Bianchi et al., 2018). The authors posit that individuals might negatively judge those who experience burnout (as happens with those suffering from numerous illnesses including depression and anxiety, see Bharadwaj et al., 2017).

This study also sought to construct a burnout stigma measure applicable to U.S. populations. The measure was constructed from items and themes modified from established stigma and burnout scales (see description in section Materials and Methods). Sampling and evaluation of these new items was completed in both academic and occupational samples. Based on prior findings, burnout stigma was expected to be of appreciative magnitude in both populations. In sum, Study 1 evaluated support for the content validity of burnout stigma and provided initial data on a novel perceived burnout stigma scale that can be used in both occupational and school settings.

### Materials and Methods

#### Participants

For the occupational sample, 144 employed adults (*M*_age_ = 34.50, *SD* = 9.85 years, Males = 65.2%) completed the measure using Amazon Mechanical Turk. Eligibility criteria included at least 30 h of weekly work/130 h monthly work. Work sample demographics include: 74% Caucasian, 8% African American, 11% Asian, 5% Hispanic, and 2% endorsed either biracial or non-disclosed ethnicity. For the student sample, 174 undergraduate students completed the measure in an online survey (*M*_age_ = 19.21, *SD* = 1.12 years, Females = 93%). Eligibility criteria include completing a full semester of college. Student demographics include: 70% Caucasian, 15% African American, 2% Asian, 9% Hispanic, and 4% endorsed biracial/non-disclosed ethnicity with 20% Freshmen, 33% Sophmore, 25% Junior, and 22% Senior.

#### Measures

##### Burnout and burnout stigma beliefs

Several items were used to assess student beliefs about burnout and burnout stigma. One question was: “Have you heard of the term ‘school burnout’?” with three response options (1 = No, never, 2 = Maybe but unsure, 3 = Yes). Subsequent questions were, “Do you think you suffer negative outcomes because of school burnout?” “Do you think school burnout is dangerous to one’s health?” “Do you think the university should provide support for students suffering from school burnout?” and “Do you think those suffering from school burnout may be stigmatized?” These questions were answered on a 4-point scale (1 = No, not at all, 2 = A little, 3 = Moderately so, 4 = Yes, definitely).

##### Burnout stigma

The burnout stigma measure was constructed with items that used phrasing and themes from previously established mental health stigma and burnout scales. These included the Stigma Scale for Receiving Psychological Help, the Self-Stigma of Mental Illness Scale, the Maslach Burnout Inventory-General Survey, and the Maslach Burnout Inventory-Student Survey (Schaufeli et al., 1996, 2002; Komiya et al., 2000; Tucker et al., 2013). Relevant themes were extracted from existing descriptions of burnout and of mental health stigma and then items were created with combinations of burnout and stigma descriptors. Item stems referencing the themes of emotional exhaustion, cynicism toward one’s work, and doubt in professional efficacy were combined with references to negative or less desirable traits/behaviors/outcomes (e.g., lazy, lack of worth, poor performance, unintelligent, character flaws) to represent perceived burnout stigma items. Scale items appear in Table 1. Phrasing was adapted to fit occupational and school settings, for instance “work” was replaced with “schoolwork” where appropriate.

**TABLE 1:** Means and standard deviations for items of the burnout stigma instrument (BSI) for the student and occupational samples.

**Item**	**Students *M* (*SD*)**	**Workers *M* (*SD*)**
People who are burnt out are lazy.*	3.15 (1.71)	2.52 (1.57)
People who claim to be burnt out should work harder.	3.14 (1.64)	2.66 (1.90)
Those who feel overwhelmed by schoolwork are weak.	2.77 (1.69)	2.62 (1.62)
Those who don’t have energy for schoolwork aren’t pushing themselves enough.	3.11 (1.77)	2.92 (1.76)
People who are too emotionally exhausted to do well at school don’t deserve achievement or praise.	2.63 (1.64)	2.92 (1.81)
Those who lose interest in their schoolwork are incapable of performing well.	2.91 (1.64)	3.09 (1.76)
People who question why their schoolwork is important are not worth the investment of time and resources.	2.83 (1.63)	2.72 (1.57)
People who think their schoolwork is pointless wouldn’t make good friends.*	2.84 (1.69)	2.60 (1.64)
Those who feel inadequate at school are unintelligent.	2.44 (1.57)	2.44 (1.62)
People who are burnt out have some character flaw.	2.50 (1.61)	2.47 (1.68)

*Item eliminated via factor analysis.

Participants were asked, “Please rate the degree to which most other people would agree with the following statements. Your responses should reflect your impression of others’ beliefs and not necessarily your own. Most people believe that.” which was then followed by the stigma items and response scale. Ten items were included as short survey lengths yield higher response rates (Deutskens et al., 2004). Responses were given on a 7-point scale (1 = Strongly Disagree, Moderately Disagree, Slightly Disagree, Neither Agree Nor Disagree, Slightly Agree, Moderately Agree, 7 = Strongly Agree).

#### Procedure

Data collection from all eligible participants was completed via an online survey which contained demographics and the burnout stigma scale. The occupational sample was collected via Amazon Mechanical Turk and paid $2 (USD). The student sample was recruited from undergraduate classrooms as an option for voluntary class credit. Extra credit was generally less than 1% of the final grade. Student data were collected in the middle (weeks 3–9) of the spring academic semester. All participants gave their written consent prior to study participation and approval was obtained from the institutional review board before any data were collected.

### Results and Discussion

#### Student Sample

**FIGURE 1:**
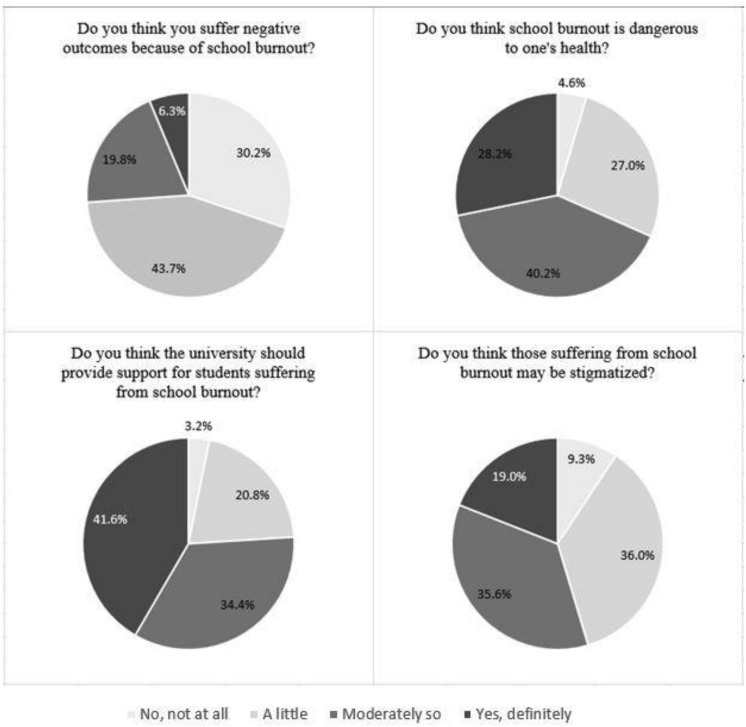
Responses to items exploring the construct validity of school burnout and burnout stigma.

First, beliefs about burnout within an academic context, “school burnout,” and perceived burnout stigma were evaluated. A minority of students (10.2%) had never heard of the term school burnout and approximately a quarter (27.2%) reported “maybe but unsure” (Figure 1). However, most students (62.6%) had heard of the term school burnout, which suggests school burnout is a salient problem visible to students. Furthermore, over 50% of students reported that those suffering from school burnout may be stigmatized and that the university should provide support for these students.

Next, regarding perceived burnout stigma, exploratory factor analysis was conducted. Means and standard deviations of items are reported in Table 1. Skewness of items ranged from 0.32 to 0.89 (*SE* = 0.10) and Kurtosis ranged from -0.16 to -0.99 (*SE* = 0.20). Exploratory factor analysis (EFA) of the 10-burnout stigma items yielding a dominant first factor with an eigenvalue of 7.05. The next largest eigenvalue was 0.83. All items demonstrated strong factor loadings, ranging from 0.660 to 0.889 with good scale reliability (α = 0.95). Principal components factor analysis using varimax rotation demonstrated a single factor (67.40% of the variance). Initial model fit indices conducted with structural equation model (SEM) via Mplus (Version 8) utilizing all ten items loading onto a single latent factor revealed marginal model fit: χ^2^(36) = 92.11, *p* < 0.001, CFI = 0.87, TLI = 0.88, SRMR = 0.066. After examination of modification indices, items 1 and 8 were removed, and a covariation between items 6 and 7 was added. Fit indices then demonstrated acceptable (good) model fit: χ^2^(20) = 47.03, *p* < 0.001, CFI = 0.96, TLI = 0.94, SRMR = 0.052.

#### Occupational Sample

Item descriptives for the perceived burnout stigma measure from the occupational sample are reported in Table 1. Skewness of items ranged from 0.35 to 0.98 (*SE* = 0.20) and Kurtosis ranged from 0.03 to -0.98 (*SE* = 0.40). The EFA yielded a dominant first factor with an eigenvalue of 6.75, eight times larger than the next eigenvalue (0.780) with strong factor loadings ranging from 0.686 to 0.876 producing good scale reliability (α = 0.94). Similar to the student sample, the principal components factor analysis using varimax rotation demonstrated a single factor (64% of the variance). SEM based model fit indices using all ten items set to load onto a single latent factor revealed marginal model fit: χ^2^(36) = 165.62, *p* < 0.001, CFI = 0.89, TLI = 0.87, SRMR = 0.065. After examination of modification indices, items 1 and 8 were removed, and a covariation between items 6 and 7 was added. Fit indices then indicated good model fit: χ^2^(20) = 49.94, *p* < 0.001, CFI = 0.97, TLI = 0.95, SRMR = 0.058.

Overall, perceived stigma for both school and occupational burnout appear to be appreciable (in regards to mean scale values) suggesting that burnout is perceived to be stigmatized in school and work settings. Students indicate they are aware of school burnout and its relationship to one’s health. Importantly, a sizable majority of students (71.6%) feel that school burnout may be stigmatized. The findings that burnout is known and stigmatized indicate that those who experience burnout may feel judged by their peers, and supports the view that burnout stigma is widespread. Furthermore, there appears to be a single factor representing an eight-item burnout stigma instrument (BSI-8) that can be used to assess perceived burnout stigma in both work and academic populations.

Although these findings are promising, many important questions remain. First, the factor structure identified requires confirmation using independent samples. Second, whether own burnout relates to perceptions of stigmatization needs examination as some evidence indicates that those who are burnt out are more likely to endorse perceptions of general mental health stigma than those who are not burnt out (Dyrbye et al., 2015). Third, does perceived burnout stigma serve as a risk factor for mental health?

## Study 2

This study sought to extend the examination of burnout stigma in both occupational and academic samples by confirming the factor structure of the burnout stigma measure (BSI-8) identified in Study 1 using confirmatory factor analysis. It also investigates associations between the BSI-8, burnout, and several indicators of mental health (e.g., depression, anxiety, and stress). To date, conflicting evidence exists regarding associations between burnout and stigma, and no research has explored this relationship with burnout-specific stigma. For example, Dyrbye et al. (2015) showed that in medical students increased perceptions of general mental health stigma correspond to greater burnout symptomology whereas Mullen and Crowe (2017) report a small negative correlation between these constructs in school counselors. Also, in sample of occupational mental health non-professionals residing in Japan, Mitake et al. (2019) found inconsistent relationships between burnout and perceived mental-illness-related stigma. Although investigating *self*-stigma and not *perceived* mental-illness-related stigma, a comparative study of Lithuanian and USA non-medical mental health care providers demonstrated that global MBI scores were linked to self-stigma of seeking help, but only in the Lithuanian sample (Endriulaitienė et al., 2019). The following study therefore examines the potential relationship between burnout symptomology and perceived burnout stigma in samples of workers and students. Finally, it also explores whether burnout stigma, independently of one’s burnout, serves as a risk factor for adverse mental health outcomes (self-reported depression, anxiety, and stress) in occupational and academic settings.

### Materials and Methods

#### Participants

For the occupational sample, 122 employed adults (*M*_age_ = 32.19, *SD* = 10.41 years, Males = 69.43%) participated. Eligibility criteria included working at least 30 h per week or 130 h of work per month. Work sample demographics include: 70% Caucasian, 9% African American, 9% Asian, 7% Hispanic, and 5% endorsed either biracial or non-disclosed ethnicity. For the academic sample, 500 undergraduate students (85% females, *M*_age_ = 19.57 years, *SD* = 1.77) participated. Students who completed at least one full academic semester were eligible for study participation. Sample demographics include: 65% Caucasian, 17% African American, 2% Asian, 8% Hispanic, and 8% endorsed either biracial or non-disclosed ethnicity with 18% Freshmen, 32% Sophmore, 30% Junior, and 20% Senior.

#### Measures

##### Burnout stigma

The 8-item burnout stigma instrument (BSI-8) created by the authors in Study 1 was used in Study 2. Phrasing was adapted to fit occupational and school settings, for instance “work” was replaced with “schoolwork” (α = 0.94 work sample, α = 0.96 school sample).

##### Burnout

Burnout in the occupational sample was measured with the Maslach Burnout Inventory-General Survey (MBI-GS; Schaufeli et al., 1996). The MBI-GS consists of 16 items that constitute three scales: exhaustion (5 items, α = 0.91), cynicism (5 items, α = 0.93), and professional efficacy (6 items, α = 0.89). Burnout in the academic sample was measured with the Maslach Burnout Inventory-Student Survey (MBI-SS; Schaufeli et al., 2002). The MBI-SS consists of 15 items that constitute three scales: exhaustion (5 items, α = 0.92), cynicism (4 items, α = 0.93), and professional efficacy (6 items, α = 0.92). Items include, “I feel emotionally drained by my studies,” “I have become less enthusiastic about my studies,” and “I can effectively solve the problems that arise in my studies,” for exhaustion, cynicism, and professional efficacy, respectively. Both MBIs use a 7-point frequency rating (0 = never to 6 = everyday). Higher scores on exhaustion and cynicism and low scores on efficacy are indicative of greater burnout. MBI efficacy scores were reverse coded to compute composite scores. Summed subscale scores yielded an overall burnout score, with higher scores indicating greater burnout.

##### Mental health indicators

Indicators of mental health were measured with The Depression, Anxiety and Stress Scale-21 Items (DASS-21; Lovibond and Lovibond, 1995). The DASS-21 is a self-report measure of three scales designed to measure the emotional states of depression, anxiety, and stress. Participants are asked to read over statements and indicate how much the statement applied to them over the past week (0 = did not apply to me, 3 = applied to me very much or most of the time). Each of the DASS-21 subscales contains 7 items with composite subscale scores for depression (DASS-D), anxiety (DASS-A), and stress (DASS-S) being calculated by summing the scores for the relevant items. Higher scores equate to higher symptomology. Reliability was high with α > 0.95 for all the subscales in both samples.

#### Procedure

As in Study 1, data were obtained using an online survey. The occupational sample was recruited using Amazon Mechanical Turk and paid $2 (USD). The student sample came from undergraduate classrooms as an option for voluntary extra class credit. Extra credit was generally less than 1% of the final grade. Student data were collected in the middle (weeks 3–9) of the spring academic semester. All participants gave written consent prior to participation and the institutional review board approved the study before data collection.

#### Statistical Analyses

Confirmatory factor analyses (CFA) of the burnout stigma scale model as identified in Study 1 (unidimensional loading of 8 items and allowing items 6 and 7 to covary) were conducted via structural equation modeling (SEM) in Mplus (Version 8) using robust maximum likelihood estimation. CFAs were done independently for the occupational and academic samples. Hu and Bentler’s (1999) recommendations informed evaluation of model fit, which is considered good when chi-square is non-significant, CFI and TLI approximate or are greater than 0.95, and SRMR is below 0.08. Pearson correlations examined the bivariate relationships between burnout stigma, burnout (global scores from the MBI-GS in the occupational sample and the MBI-SS in the academic sample), and the depression, anxiety, and stress subscales of the DASS-21. Three hierarchical multiple regressions (HMR) were conducted controlling for burnout to evaluate the unique contribution of burnout stigma in predicting variance in depression, anxiety, and stress scores.

### Results and Discussion

Specifying a single factor and allowing covariance between items 6 and 7 yielded a good model fit in both samples: occupational sample, χ^2^(20) = 48.17, *p* < 0.001, CFI = 0.94, TLI = 0.93, SRMR = 0.029; academic sample, χ^2^(20) = 44.70, *p* < 0.001, CFI = 0.96, TLI = 0.95, SRMR = 0.019. Burnout stigma was not related to burnout in either the occupational (*r* = 0.03) or academic (*r* = 0.06) samples. Burnout stigma, however, was related to all three DASS-21 subscales in the occupational sample and academic samples (*p* < 0.05, see Table 2).

**TABLE 2:** Correlation matrices of stigma, burnout, and DASS-21 in the work and student samples.

**Variable**	***M* ± *SD***	**1**	**2**	**3**	**4**	**5**

**Work Sample**						
1. Burnout stigma	21.40 ± 10.97	1.00	0.03	0.23^∗^	0.30^∗∗^	0.24^∗∗^
2. MBI-GS	40.45 ± 18.52		1.00	0.70^∗∗^	0.59^∗∗^	0.70^∗∗^
3. DASS-D	7.64 ± 10.08			1.00	0.81^∗∗^	0.82^∗∗^
4. DASS-A	6.28 ± 9.46				1.00	0.87^∗∗^
5. DASS-S	8.77 ± 9.01					1.00

**Student Sample**						

1. Burnout stigma	22.56 ± 11.69	1.00	0.06	0.15^∗∗^	0.15^∗∗^	0.16^∗∗^
2. MBI-SS	44.21 ± 12.06		1.00	0.46^∗∗^	0.35^∗∗^	0.39^∗∗^
3. DASS-D	5.21 ± 4.65			1.00	0.72^∗∗^	0.73^∗∗^
4. DASS-A	5.99 ± 4.98				1.00	0.79^∗∗^
5. DASS-S	6.41 ± 4.33					1.00

N = 122 for occupational sample. N = 500 for academic sample. MBI-GS, Maslach Burnout Inventory; DASS-D, Depression Anxiety Stress Scale – Depression, DASS-A, Depression Anxiety Stress Scale – Anxiety; DASS-S, Depression Anxiety Stress Scale – Stress, MBI-SS, Maslach Burnout Inventory – Student Survey. *p < 0.05, **p < 0.01, two-tailed.

In the occupational sample, HMR analyses (presented in Table 3) showed that after controlling for burnout, burnout stigma accounted for additional variance in depression, Δ*F*(1, 119) = 7.73, *p* = 0.006, anxiety, Δ*F*(1, 119) = 13.34, *p* < 0.001, and stress, Δ*F*(1, 119) = 9.61, *p* = 0.002. Burnout stigma uniquely predicted 3% of the variance in depression scores, 7% of the variance in anxiety scores, and 4% of the variance in stress scores. Similarly, in the academic sample, HMR analyses showed that after controlling for burnout, burnout stigma accounted for additional variance in scores of depression, Δ*F*(1, 497) = 11.13, *p* = 0.001, anxiety, Δ*F*(1, 497) = 11.93, *p* = 0.001, and stress, Δ*F*(1, 497) = 11.33, *p* = 0.001. Burnout stigma uniquely accounted for approximately 2% of the variance in scores of depression, anxiety, and stress.

**TABLE 3:** Hierarchal multiple regressions of DASS-21 scales on burnout stigma controlling for personal burnout in occupational and academic samples.

**Criterion**	**Step**	**Predictors**	**β**	***p***	**Model *R*^2^**	**Model Δ*R*^2^**	**Model *F***

**Work**							
DASS-D	S1	MBI-GS	0.70	0.000	0.49		*F*(1, 120) = 113.50, *p* < 0.000
	S2	MBI-GS	0.69	0.000	0.52		
		Stigma	0.18	0.006		0.03	Δ*F*(1, 119) = 7.73, *p* = 0.006
DASS-A	S1	MBI-GS	0.59	0.000	0.34		*F*(1, 120) = 62.79, *p* < 0.001
	S2	MBI-GS	0.57	0.000	0.41		
		Stigma	0.26	0.000		0.07	Δ*F*(1, 119) = 13.34, *p* < 0.001
DASS-S	S1	MBI-GS	0.70	0.000	0.49		*F*(1, 120) = 116.66, *p* < 0.001
	S2	MBI-GS	0.69	0.000	0.53		
		Stigma	0.20	0.002		0.04	Δ*F*(1, 119) = 9.61, *p* = 0.002

**Student**							

DASS-D	S1	MBI-SS	0.46	0.000	0.23		*F*(1, 498) = 150.08, *p* < 0.000
	S2	MBI-SS	0.45	0.000	0.25		
		Stigma	0.15	0.001		0.02	Δ*F*(1, 497) = 11.13, *p* = 0.001
DASS-A	S1	MBI-SS	0.35	0.000	0.13		*F*(1, 498) = 87.45, *p* < 0.001
	S2	MBI-SS	0.35	0.000	0.15		
		Stigma	0.15	0.001		0.02	Δ*F*(1, 497) = 11.93, *p* = 0.001
DASS-S	S1	MBI-SS	0.39	0.000	0.15		*F*(1, 498) = 100.62, *p* < 0.001
	S2	MBI-SS	0.39	0.000	0.17		
		Stigma	0.15	0.001		0.02	Δ*F*(1, 497) = 11.33, *p* = 0.001

*N = 122 for occupational sample. N = 500 for academic sample. MBI-GS, Maslach Burnout Inventory; DASS-D, Depression Anxiety Stress Scale – Depression; DASS-A, Depression Anxiety Stress Scale – Anxiety; DASS-S, Depression Anxiety Stress Scale – Stress, MBI-SS, Maslach Burnout Inventory – Student Survey.*

Overall, these findings confirm the factor structure of the perceived burnout stigma measure initially produced in Study 1. Extending the examination of this measure of stigma to associations with covariates showed that, contrary to prior research (Dyrbye et al., 2015; Mullen and Crowe, 2017), burnout stigma was not related to one’s own burnout in either sample. These discrepant findings may reflect differences in the populations sampled as well as other important methodological differences. For example, Dyrbye et al. (2015) used only the emotional exhaustion and cynicism MBI subscales which were then categorized into low, medium, and high symptomologies. Furthermore, via chi-square tests, burnout relationships were evaluated with a stigma endorsement measure that was unstandardized and not previously examined (i.e., author created questions, self-stigma vs. public stigma vs. treatment stigma questions mixed, and no factor structure examination). Lastly, previous research measured general mental health stigma and not stigma that is specific to burnout (Dyrbye et al., 2015; Mullen and Crowe, 2017). Thus, given these differences in samples and measurement, further research needs to explore any potential association between burnout and burnout stigma.

Finally, this study demonstrated that perceived burnout stigma was associated with negative affect (symptoms of depression, anxiety, and stress). The link between burnout stigma and negative affect symptoms was especially robust as it occurred in both the occupational and academic samples. These relationships also held after controlling for one’s burnout. Thus, even though this study provided evidence on the reliability of the factor structure and some evidence of the validity of the burnout stigma measure, concerns regarding direction of effect arise. Study 3 attempts to address such concerns.

## Study 3

Although Study 2 showed that burnout stigma is related to mental health (i.e., depression, anxiety, and stress scores), causal inferences are limited by the cross-sectional nature of the data. This study therefore sought to determine the temporal ordering of perceived burnout stigma and mental health indicators using two waves of data collected 6 weeks apart in a student sample. Cross-lagged stability models and bidirectional analyses were used to examine temporal relationships. Cross-lagged stability models allow examination of longitudinal relationships between variables while also controlling for their stability by having each Time 2 variable simultaneously regressed on each Time 1 variable. The occurrence of a significant cross-lagged effect reflects a relationship beyond that which can be accounted for by the stability of the constructs and their association at Time 1. The presence of bidirectional or synchronous effects between perceived burnout stigma and mental health indicators were also examined in non-recursive models.

### Materials and Methods

#### Participants

Undergraduate students (*n* = 682; 92% Females, *M*_age_ = 20.03, *SD* = 1.89 years) completed an online survey at two time points 6 weeks apart. Eligibility criteria include completing a full semester of college. Student demographics include: 69% Caucasian, 10% Black, 14% Hispanic, 2% Asian, and 5% endorsed other with 24% Freshmen, 29% Sophmore, 24% Junior, and 23% Senior.

#### Measures

##### Burnout stigma

The BSI-8 created by the authors was again used with phrasing adapted to reflect the school settings (α = 0.95 at Time 1, α = 0.96 at Time 2).

##### Mental health indicators

The Depression, Anxiety and Stress Scale (DASS-21; Lovibond and Lovibond, 1995) served as an indicator of mental health in this study. Reliability was high with α > 0.93 for all the subscales at both time waves. As Study 2 showed high intercorrelations among the subscales and similar relationships between the subscales and burnout stigma, the global composite score was used for the main analyses of this study by summing all DASS-21 items.

### Results and Discussion

**FIGURE 2:**
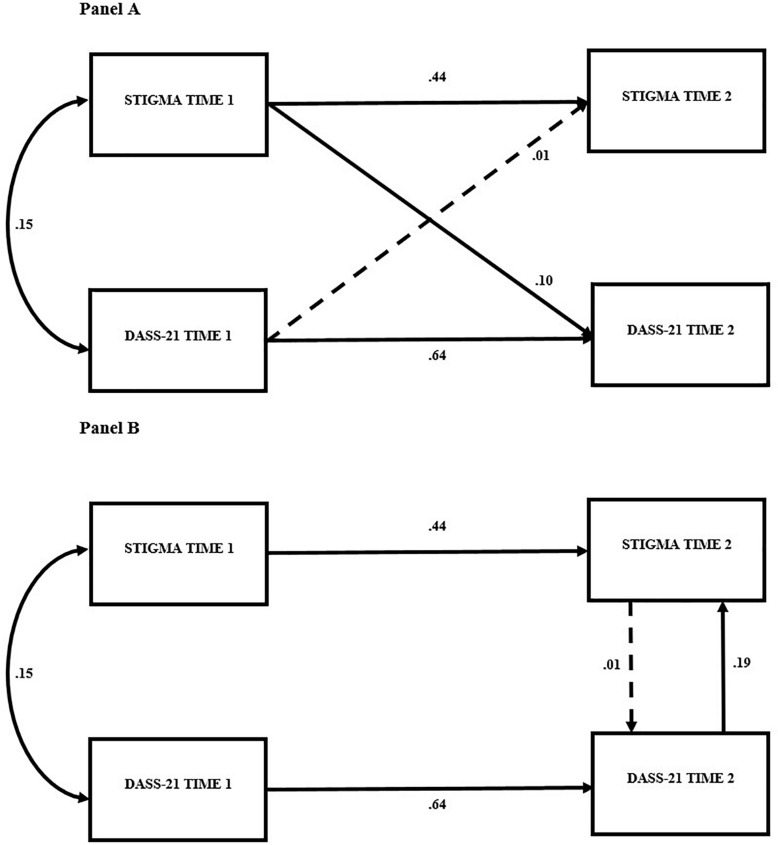
Cross-lagged models in **(A)**. Non-recursive models in **(B)**. DASS-21, Depression, Anxiety and Stress Scale. *p* < 0.05 for all coefficients on solid lines.

Examination from the cross-lagged stability models demonstrated that the effect from Time 1 burnout stigma to Time 2 DASS-21 was significant, β = 0.10, *p* < 0.05, but the effect from Time 1 DASS-21 to Time 2 burnout stigma was not, β = 0.01, ns (Figure 2A). To examine the possible bidirectional (synchronous) effects between the two indices, non-recursive models were estimated. In order to identify a bidirectional effects model, several conditions need to first be satisfied. The present model satisfies these conditions in that earlier measures of perceived burnout stigma and DASS-21 scores are presumed to be predetermined variables and thereby uncorrelated with the disturbance terms in both Time 2 equations and both cross-lagged effects are constrained to be zero. These analyses produced findings that were consistent with the results obtained in the cross-lagged stability models. Again, in each model (Figure 2B), the effect from stigma to DASS-21 was significant (β = 0.19, *p* < 0.05) but the effect in the opposite direction was not (β = 0.01, ns). It should be noted that analyses substituting DASS subscales for the global scores yielded similar results, however, for ease of presentation they are not reported here.

These findings indicate that perceived burnout stigma predicted indicators of mental health, specifically symptoms of negative affect, 6 weeks later. Taken together, the results provide evidence that perceived burnout stigma may negatively influence indicators of mental health and that this relationship is not bidirectional. This research is the first to show that perceived burnout stigma can signal issues pertaining to mental health. While these data are not experimental, it is reasonable to conclude that they support further study of a potential causal relationship. Although these findings are instructive, it should be noted that differing temporal lags (e.g., time intervals) may improve (or weaken) predictive strength.

Regarding assessment utility of the BSI-8, it would be advantageous to demonstrate the measure as potentially robust to sample and response pattern differences. One way to help do this is to use modern test theory, specifically item response theory (IRT; Hambleton et al., 1991), and supplement the classical test theory (CTT) approaches used in the prior studies. Study 4 therefore provides an IRT evaluation of the BSI-8.

## Study 4

Classical test theory (CTT) relies on correlational techniques like Cronbach’s alpha coefficients, exploratory factor analysis, and confirmatory factor analysis. Although CTT has advantages (e.g., ease of interpretation, requires smaller sample sizes, less stringent statistical assumptions) and can be effective at creating internally consistent scales, item response theory (IRT) augments the limitations of CTT by providing a more in-depth analysis of item properties (see Rusch et al., 2017 for limitations of CTT and advantages of IRT). IRT refers to a group of latent trait models such as Rasch models and rating scale models that give rise to useful techniques, such as item information analysis and differential item functioning that can be used to psychometrically optimize scales by increasing precision and minimizing measurement error (Drasgow and Hulin, 1990; Foster et al., 2017).

Although IRT approaches require larger samples and make stricter statistical assumptions than CTT, the item parameters they yield are – barring any differential item functioning –potentially subpopulation invariant (Embretson and Reise, 2000; for qualifications see Asun et al., 2017). This is a major advantage for the current research as this serves to produce test items and measurement scales that can function consistently across a range of samples, such as academic and work samples. Thus, item parameter invariance is a major advantage of IRT over CTT as it can allow researchers to generalize how items operate across populations. The current study evaluates burnout stigma responses via an IRT approach to measurement development.

### Materials and Methods

#### Participants

Seven hundred seventeen undergraduate students (90% females, *M*_age_ = 20.10 years, *SD* = 1.92) completed the BSI-8 via an online survey. Students who completed at least one full academic semester were eligible for study participation. Sample demographics include: 68% Caucasian, 11% African American, 4% Asian, 15% Hispanic, and 2% endorsed either biracial or non-disclosed ethnicity with 15% Freshmen, 35% Sophmore, 32% Junior, and 18% Senior.

#### Procedure

Participants were undergraduates from classrooms offering an option for voluntary class credit by completing an online survey. Extra credit was generally less than 1% of the final grade. Student data were collected in the middle (weeks 3–9) of the academic semester. All participants gave their written consent prior to study participation and the institutional review board approved the study before any data were collected.

#### Statistical Analysis

Item response theory analysis was conducted using the graded response model (GRM; Samejima, 1969). The GRM models how well an item differentiates between similar people, via the discrimination parameter (α), and how severe a person’s stigma toward burnout must be in order to endorse a given response level to an item, via the threshold parameters (β); each item has 6 threshold parameters, corresponding to the number of response options (*k* = 7) minus 1. Like all IRT model parameters, these are interpreted relative to theta (θ), which is a person’s location on the latent trait continuum. For this scale, higher levels of theta correspond to more severe burnout stigma. Theta scores are normally distributed with *M* = 0 and *SD* = 1.

### Results and Discussion

**FIGURE 3:**
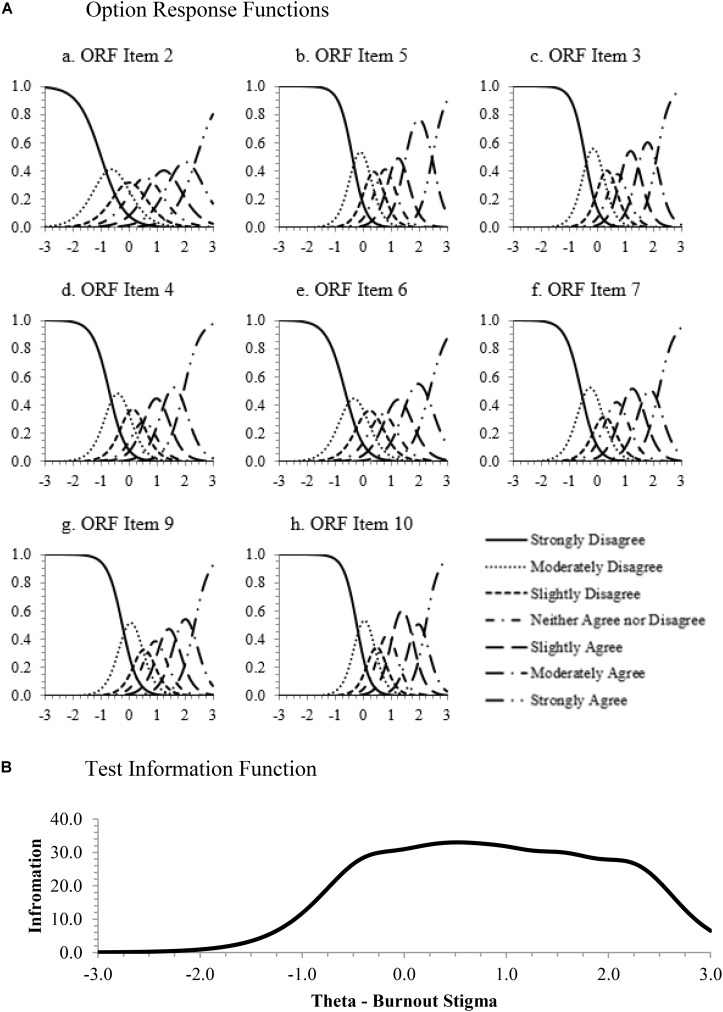
Item response theory graphs. **(A)** Option response functions. **(B)** Test information function.

Principal components analysis showed that the data met the requirement of sufficient unidimensionality (Reckase, 1979). The unidimensional model was appropriate based on a predominant first factor explaining 73% of the variance. Item parameters and fit statistics are shown in Table 4. All items demonstrated good psychometric properties. The model-data fit was acceptable, as indicated by χ^2^/df ratios less than three (LaHuis et al., 2011). The item discrimination parameters show good differentiation among responses, leading to high levels of item information (i.e., accurate information about an individual’s level of burnout stigma). The threshold parameters are spread out evenly across normally distributed latent continua, leading to good item information at all levels of burnout stigma. This is easily seen via option response functions (ORFs) that show the relation between an individual’s level of burnout stigma, labeled “Theta – Burnout Stigma” on the abscissa, and the probability of responding to that item with a given level of endorsement (i.e., *Strongly Disagree* to *Strongly Agree*) on the ordinate. Figure 3A displays ORFs from each scale item. Each trace line represents the probably of endorsing the item at a specific level based on the person’s amount of stigma toward burnout. The smooth lines identify peaks for each response option, and coverage of the full continuum all reflect ideal ORF characteristics.

**TABLE 4:** Grade response model parameter estimates and fit statistics.

**Item**	**α**	**β_1_**	**β_2_**	**β_3_**	**β_4_**	**β_5_**	**β_6_**	**S-χ^2^/df**
2	1.41	−1.01	−0.28	0.27	0.89	1.59	2.42	1.74
3	2.52	−0.45	0.14	0.53	0.90	1.47	2.12	2.06
4	1.99	−0.73	−0.11	0.35	0.69	1.26	1.95	2.49
5	2.45	−0.40	0.18	0.57	0.99	1.50	2.47	1.94
6	1.77	−0.66	−0.03	0.47	0.94	1.56	2.38	1.91
7	2.10	−0.60	0.06	0.43	0.93	1.57	2.19	1.45
9	2.20	−0.25	0.36	0.71	1.15	1.69	2.34	2.68
10	2.53	−0.24	0.31	0.64	1.07	1.72	2.24	2.00

Figure 3B models the test information function (TIF) for the full scale. Higher levels of information result in more accurate person score estimates and, therefore, lower levels of error in the estimate of an individual’s level of stigma toward burnout whether items are scored using IRT or traditional sum or average methods. The TIF shows high levels of information for a majority of the trait range from θ = -0.5 to θ = 2.5, indicating that the scale does a good job scoring people who have average to high levels of stigma against burnout but is less efficacious for scoring those very low in stigma. This may be interpreted as less problematic, especially if the scale is used in clinical settings, as mental health providers may be less concerned with identification of those who do not have higher stigma scores.

In regard to measurement efficiency, the location of the test information is determined by the location of the items’ threshold parameters. As there is a good deal of overlap in the threshold locations, future research could investigate the utility of a short form version of this scale consisting of three to four items. The short form scale then would be useful for repeated measurement in longitudinal or experience sampling research.

In sum, the results of the IRT analysis validate the excellent psychometric properties of the eight-item burnout stigma instrument (BSI-8) by showing consistently high information across a large range of the latent continuum, good model-data fit, high item discrimination parameters, and equally spaced item threshold parameters. These results corroborate the good psychometric properties demonstrated in the exploratory and confirmatory factor analyses conducted earlier and lay a solid foundation for additional substantive construct validation.

## General Discussion

The current research establishes a novel measure of perceived burnout stigma. Over four studies using occupational and academic samples and utilizing measurement development approaches derived from both classical test theory and item response theory, an eight-item burnout stigma instrument (BSI-8) predictive of indicators of mental health emerged that demonstrated excellent psychometric properties. As documented in an extensive literature, burnout is a prevalent and burdening condition linked to a myriad of negative outcomes affecting the well-being of diverse populations. Importantly, stigma is one significant barrier to help-seeking behaviors. The present studies highlight the presence of appreciable levels of perceived burnout stigma in students enrolled in university studies as well as individuals holding vocational positions in the workforce.

This work built on Bianchi et al. (2016) by providing more support to show that burnout is viewed as a stigmatized condition. The current work found higher levels of burnout stigma than previous research, which could reflect the use of differing instruments across studies (Bianchi et al., 2016). While previous research explored the stigma level of burnout as compared to depression (Mendel et al., 2015; Bianchi et al., 2016), this research is the first to show that burnout stigma can signal issues pertaining to mental health, particularly negative affect.

The link between the stigmatization of burnout and mental health issues is not surprising, as other forms of mental health stigma predict depression (Pyle et al., 2015; Lee et al., 2017). The relationship between burnout stigma and mental health indicators supports the authors’ prediction that burnout stigma may affect individuals similarly to other forms of mental health stigma. School and work-based policies should discuss burnout stigma and efforts should be made to decrease its effects. This is reflected in the current data as students felt the university should play a role in helping combat burnout. In order to ameliorate the effects of perceived burnout stigma, interventions may seek to improve social support and encourage positive coping strategies for people at risk for burnout (Chronister et al., 2013).

Burnout stigmatization can be understood through the lens of social-cognitive stigma theory. According to this model, society creates stereotypes about specific groups, which leads to prejudice and discrimination (Corrigan, 2018). In the case of burnout, participants endorsed items that aligned with the stereotype that people who are burnt out are somehow at fault for their condition and should work harder to meet societal demands. Thus, codifying the types of prejudice and discrimination that may result from burnout symptomology is important in academic settings, the work place, and in counseling. For instance, it is possible that the struggles of burnt out individuals will be dismissed, or more negatively evaluated by others, including peers, teachers and bosses. Counselors should apply this knowledge to their work with students and employed adults who experience burnout. They might consider applying cognitive-restructuring techniques to address directly the stigma faced by their clients (Hays, 2009).

In order to more comprehensively understand perceived burnout stigma and the more nuanced ways stigmatization may affect people, future research may find it fruitful to explore the phenomenological experiences of burnt out individuals. A qualitative approach examining the accounts of such individuals would help supplement, improve, and further validate the instrument created from this research. Including individuals with lived experience is a central component when studying stigmatized groups (Corrigan, 2018). Community-based participatory research is a useful method for this process. This method involves working with affected individuals in a collaborative way that allows them to drive the research based on lived experiences. The current project attempted to explore perceived burnout stigma utilizing previously established conceptualizations of the construct, and the BSI-8 may be further evaluated using qualitative methodologies by those who experience burnout.

Future research may also explore potential differences in burnout stigma constructs. Perceived burnout stigma, which was the focus of the current research, and internalized (self) burnout stigma may operate differently. Both perceived stigma and self-stigma measure stereotyped attitudes that are influenced by cultural, historical, and situational factors (Dovidio et al., 2000). Thus, an individual’s perception of public stigma and how they personally internalize the stigma may differentially predict help seeking behaviors, stress appraisals, physiological reactivity, and so on. However, self-stigma may be a stronger predictor of mental health than perceived stigma, and several factors such as support and coping styles may explain why perceived stigma is internalized as self-stigma in certain people (Chronister et al., 2013). While this study explored perceived burnout stigma as a first step toward understanding the public stigmatization of this construct, future research is needed to develop measures of both perceived and self-burnout stigma and evaluate their relationship to burnout.

Notwithstanding the importance of this research, its limitations deserve attention. First, the samples studied were limited; the student samples came from only one university and were predominately Caucasian and female (although U.S. universities are comprised of more females than males, U. S. Department of Education, National Center for Education Statistics, 2017) and the occupational samples were collected using the Amazon Turk platform. Additional and more varied sampling is necessary (including expanding sampling regarding socioeconomic status, ethnicity, gender, and culture), especially regarding inferences pertaining to covariate associations. However, use of the IRT approach does help buffer against potential sub-population differences in the response structure corresponding to the burnout stigma items. Relatedly, only one measure (DASS-21) served to represent negative affect in this research. Future research could examine how burnout stigma predicts more clinically diagnostic measures of mental illness (e.g., Beck Depression Inventory) as well as attitudes and behaviors more directly related to health seeking behaviors (see Mullen and Crowe, 2017 for a potential causal framework).

Another issue deserving future attention is the relationship between burnout stigma and depression stigma. While the orthogonality of burnout and depression and the potential causal relationship between them are still being evaluated, burnout and depression-related stigma differ in how they are socially understood and represented (Bianchi et al., 2016, 2018). Continued research should explore the different “groupness” qualities for depression vs. burnout (Corrigan, 2018). As this research is the first to report on perceived burnout stigma in the U.S., research into how these differing forms of stigma uniquely relate to mental health challenges or other negative outcomes might be fruitful. Findings suggest that combatting perceived burnout stigma may help lead those suffering from mental health challenges to avenues supportive of health treatment.

In summary, this research provides evidence that burnout is stigmatized in academic and occupational contexts and indicates that burnt out individuals may be at risk for the negative mental health effects of stigma. This research provides preliminary data on a new instrument to measure perceived burnout stigma which can be used by psychologists, counselors, and researchers to evaluate the impact of this phenomenon and explore potential interventions to prevent negative outcomes.

## Data Availability Statement

The datasets generated for this study are available on request to the corresponding author.

## Ethics Statement

The studies involving human participants were reviewed and approved by Florida State University Office for Human Subjects Protection. The patients/participants provided their written informed consent to participate in this study.

## Author Contributions

RM and JT contributed to the conception of the topic and the first draft of the manuscript. RM designed the studies. RM and GF performed the statistical analyses. GS and FF wrote sections of the manuscript. All authors contributed to manuscript revisions and approved the submitted version.

## Conflict of Interest

GF was employed by the company Maritz. The remaining authors declare that the research was conducted in the absence of any commercial or financial relationships that could be construed as a potential conflict of interest.

## References

[B1] Asun R. A. Rdz-Navarro K. Alvarado J. M. (2017). The sirens’ call in psychometrics: the invariance of IRT models. *Theory Psychol.* 27 389–406. 10.1177/0959354317706272

[B2] Bakker A. B. Le Blanc P. M. Schaufeli W. B. (2005). Burnout contagion among intensive care nurses. *J. Adv. Nurs.* 51 276–287. 10.1111/j.1365-2648.2005.03494.x 16033595

[B3] Beck F. Guignard R. Rolland, Du Roscoat E. Briffault X. (2009). “Attitudes et opinions vis-à-vis de la dépression,” in *La Dépression en France (Enquête Anadep)* 2005, eds Chan Chee C. Beck F. Sapinho D. Guilbert P. (Saint-Denis: INPES), 119–140.

[B4] Bharadwaj P. Pai M. M. Suziedelyte A. (2017). Mental health stigma. *Econ. Lett.* 159 57–60.

[B5] Bianchi R. Boffy C. Hingray C. Truchot D. Laurent E. (2013). Comparative symptomatology of burnout and depression. *J. Health Psychol.* 18 782–787. 10.1177/1359105313481079 23520355

[B6] Bianchi R. Rolland J. Salgado J. F. (2018). Burnout, depression, and borderline personality: a 1,163-participant study. *Front. Psychol.* 8:2336 10.3389/fpsyg.2017.02336 PMC5769336 29375447

[B7] Bianchi R. Schonfeld I. S. Laurent E. (2019). Burnout: moving beyond the status quo. *Int. J. Stress Manage.* 26 36–45. 10.1037/str0000088

[B8] Bianchi R. Verkuilen J. Brisson R. Schonfeld I. S. Laurent E. (2016). Burnout and depression: label-related stigma, help-seeking, and syndrome overlap. *Psychiatry Res.* 245 91–98. 10.1016/j.psychres.2016.08.025 27529667

[B9] Busby Grant J. Bruce C. P. Batterham P. J. (2016). Predictors of personal, perceived and self-stigma towards anxiety and depression. *Epidemiol. Psychiatric Sci.* 25 247–254. 10.1017/S2045796015000220 PMC6998700 25791089

[B10] Cechnicki A. Matthias C. Angermeyer A. B. (2011). Anticipated and experienced stigma among people with schizophrenia: its nature and correlates. *Soc. Psychiatry Epidemiol.* 46 643–650. 10.1007/s00127-010-0230-2 20495975

[B11] Chronister J. Chou C. C. Liao H. Y. (2013). The role of stigma coping and social support in mediating the effect of societal stigma on internalized stigma, mental health recovery, and quality of life among people with serious mental illness. *J. Commun. Psychol.* 41 582–600. 10.1002/jcop.21558

[B12] Corrigan P. W. (2018). Defining the stereotypes of health conditions: methodological and practical considerations. *Stigma Health* 3 131–138. 10.1037/sah0000085

[B13] Crisp A. Gelder M. Goddard E. Meltzer H. (2005). Stigmatization of people with mental illnesses: a follow-up study within the Changing Minds campaign of the Royal College of Psychiatrists. *World Psychiatry* 4:106. PMC1414750 16633526

[B14] Deligkaris P. Panagopoulou E. Montgomery A. J. Masoura E. (2014). Job burnout and cognitive functioning: a systematic review. *Work Stress* 28 107–123. 10.1080/02678373.2014.909545

[B15] Deutskens E. De Ruyter K. Wetzels M. Oosterveld P. (2004). Response rate and response quality of internet-based surveys: an experimental study. *Market. Lett.* 15 21–36. 10.1023/b:mark.0000021968.86465.00

[B16] Dovidio J. F. Major B. Crocker J. (2000). “Stigma: introduction and overview,” in *The Social Psychology of Stigma*, eds Heatherton T. F. Kleck R. E. Hebl M. R. Hull J. G. Heatherton T. F. Kleck R. E. (New York, NY: Guilford Press), 1–28.

[B17] Drasgow F. Hulin C. L. (1990). “Item response theory,” in *Handbook of Industrial and Organizational Psychology*, eds Dunnette M. Hough L. Triandis H. (Palo Alto, CA: Consulting Psychologists Press), 577–636.

[B18] Dyrbye L. N. Eacker A. Durning S. J. Brazeau C. Moutier C. Massie F. S. (2015). The impact of stigma and personal experiences on the help-seeking behaviors of medical students with burnout. *Acad. Med.* 90 961–969. 10.1097/acm.0000000000000655 25650824

[B19] Embretson S. E. Reise S. P. (2000). *Item Response Theory.* Mahwah, NJ: Lawrence Erlbaum Associates.

[B20] Endriulaitienė A. Žardeckaitė-Matulaitienė K. Pranckevičienė A. Markšaitytė R. Tillman D. R. Hof D. D. (2019). Self-Stigma of seeking help and job burnout in mental health care providers: the comparative study of Lithuanian and the USA samples. *J. Workplace Behav. Health* 34 129–148. 10.1080/15555240.2019.1586549

[B21] Foster G. C. Min H. Zickar M. J. (2017). Review of item response theory practices in organizational research: lessons learned and paths forward. *Organ. Res. Methods* 20 465–486. 10.1177/1094428116689708

[B22] Freudenberger H. J. (1974). Staff burn-out. *J. Soc. Issues* 30 159–165. 10.1111/j.1540-4560.1974.tb00706.x

[B23] Golkar A. Johansson E. Kasahara M. Osika W. Perski A. Savic I. (2014). The influence of work-related chronic stress on the regulation of emotion and on functional connectivity in the brain. *PLoS ONE* 9:e104550 10.1371/journal.pone.0104550 PMC4153588 25184294

[B24] Hambleton R. K. Swarninathan H. Rogers H. J. (1991). *Fundamentals of Item Response Theory.* Newbury Park, CA: Sage.

[B25] Hays P. A. (2009). Integrating evidence-based practice, cognitive–behavior therapy, and multicultural therapy: ten steps for culturally competent practice. *Prof. Psychol. Res. Pract.* 40 354–360. 10.1037/a0016250

[B26] Hu L. T. Bentler P. M. (1999). Cutoff criteria for fit indexes in covariance structure analysis: conventional criteria versus new alternatives. *Struct. Equat. Model. Multidiscipl. J.* 6 1–55. 10.1080/10705519909540118

[B27] Jennings K. S. Cheung J. H. Britt T. W. Goguen K. N. Jeffirs S. M. Peasley A. L. (2015). How are perceived stigma, self-stigma, and self-reliance related to treatment-seeking? A three-path model. *Psychiatr. Rehabil. J.* 38 109–116. 10.1037/prj0000138 25844914

[B28] Komiya N. Good G. E. Sherrod N. B. (2000). Emotional openness as a predictor of college students’ attitudes toward seeking psychological help. *J. Counsel. Psychol.* 47:138 10.1037/0022-0167.47.1.138

[B29] Koutsimani P. Montgomery A. Georganta K. (2019). The relationship between burnout, depression, and anxiety: a systematic review and meta-analysis. *Front. Psychol.* 10:284 10.3389/fpsyg.2019.00284 PMC6424886 30918490

[B30] LaHuis D. M. Clark P. O’Brien E. (2011). An examination of item response theory item fit indices for the graded response model. *Organ. Res. Methods* 14 10–23. 10.1177/1094428109350930

[B31] Lannin D. G. Vogel D. L. Brenner R. E. Abraham W. T. Heath P. J. (2016). Does self-stigma reduce the probability of seeking mental health information? *J. Counsel. Psychol.* 63 351–358. 10.1037/cou0000108 26323042

[B32] Lee L. Tung H. Chen S. Fu C. (2017). Perceived stigma and depression in initially diagnosed pulmonary tuberculosis patients. *J. Clin. Nurs.* 26 4813–4821. 10.1111/jocn.13837 28370819

[B33] Lovibond S. H. Lovibond P. F. (1995). *Manual for the Depression Anxiety & Stress Scales*, 2nd Edn Sydney: Psychology Foundation.

[B34] Maslach C. (2017). Finding solutions to the problem of burnout. *Consult. Psychol. J. Pract. Res.* 69 143–152. 10.1037/cpb0000090

[B35] Maslach C. Shaufeli W. B. Leiter M. P. (2001). Job burnout. *Annu. Rev. Psychol.* 52 397–422. 11148311 10.1146/annurev.psych.52.1.397

[B36] May R. W. Sanchez-Gonzalez M. A. Brown P. C. Koutnik A. P. Fincham F. D. (2014a). School burnout and cardiovascular functioning in young adult males: a hemodynamic perspective. *Stress* 17 79–87. 10.3109/10253890.2013.872618 24308407

[B37] May R. W. Sanchez-Gonzalez M. A. Fincham F. D. (2014b). School burnout: increased sympathetic vasomotor tone and attenuated ambulatory diurnal blood pressure variability in young adult women. *Stress* 18 1–9. 10.3109/10253890.2014.969703 25256608

[B38] May R. W. Seibert G. S. Sanchez-Gonzalez M. A. Fincham F. D. (2016). Physiology of school burnout in medical students: hemodynamic and autonomic functioning. *Burnout Res.* 3 63–68. 10.1016/j.burn.2016.05.001

[B39] May R. W. Seibert G. S. Sanchez-Gonzalez M. A. Fincham F. D. (2018). School burnout and heart rate variability: risk of cardiovascular disease and hyptertension in young adult females. *Stress* 21 211–216. 29382258 10.1080/10253890.2018.1433161

[B40] Mendel R. Kissling W. Reichhart T. Buhner M. Hamann J. (2015). Managers’ reactions towards employees’ disclosure of psychiatric or somatic diagnoses. *Epidemiol. Psychiatric Sci.* 24 146–149. 10.1017/s2045796013000711 PMC6998118 24308312

[B41] Milken Institute Center for the Future of Aging (2018). Ending the stress and burnout epidemic. Forbes. Available online at: https://www.forbes.com/sites/nextavenue/2018/11/26/ending-the-stress-and-burnout-epidemic/#32561e7f4b41

[B42] Mitake T. Iwasaki S. Deguchi Y. Nitta T. Nogi Y. Kadowaki A. (2019). Relationship between burnout and mental-illness-related stigma among nonprofessional occupational mental health staff. *BioMed Res. Int.* 2019 1–6. 10.1155/2019/5921703 PMC6778926 31662983

[B43] Mohammadi S. (2006). Burnout and psychological health in high school teachers. *J. Iran. Psychol.* 3 15–23.

[B44] Mullen P. R. Crowe A. (2017). Self-stigma of mental illness and help seeking among school counselors. *J. Counsel. Dev.* 95 401–411. 10.1002/jcad.12155

[B45] Oosterholt B. G. Maes J. H. Van der Linden D. Verbraak M. J. Kompier M. A. (2015). Burnout and cortisol: evidence for a lower cortisol awakening response in both clinical and nonclinical burnout. *J. Psychos. Res.* 78 445–451. 10.1016/j.jpsychores.2014.11.003 25433974

[B46] Orel E. T. (2007). Stigmatization in the long-term treatment of psychotic disorders. *Neuro Endocrinol. Lett.* 28 35–45. 17262008

[B47] Parker P. D. Salmela-Aro K. (2011). Developmental processes in school burnout: a comparison of major developmental models. *Learn. Individ. Diff.* 21 244–248. 10.1016/j.lindif.2011.01.005

[B48] Pyle M. Stewart S. K. French P. Byrne R. Patterson P. Gumley A. (2015). Internalized stigma, emotional dysfunction and unusual experiences in young people at risk of psychosis. *Early Interv. Psychiatry* 9 133–140. 10.1111/eip.12098 25775264

[B49] Ranjbar N. Ricker M. (2018). Burn Bright I: reflections on the burnout epidemic (Part one of a two-part series). *Am. J. Med.* 132 272–275. 10.1016/j.amjmed.2018.09.036 30550752

[B50] Reckase M. D. (1979). Unifactor latent trait models applied to multifactor tests: results and implications. *J. Educ. Behav. Stat.* 4 207–230. 10.3102/10769986004003207

[B51] Reis D. Xanthopoulou D. Tsaousis I. (2015). Measuring job and academic burnout with the Oldenburg Burnout Inventory (OLBI): factorial invariance across samples and countries. *Burnout Res.* 2 8–18. 10.1016/j.burn.2014.11.001

[B52] Rusch T. Lowry P. B. Mair P. Treiblmaier H. (2017). Breaking free from the limitations of classical test theory: developing and measuring information systems scales using item response theory. *Inform. Manage.* 54 189–203. 10.1016/j.im.2016.06.005

[B53] Sablik Z. Samborska-Sablik A. Drożdż J. (2013). Universality of physicians’ burnout syndrome as a result of experiencing difficulty in relationship with patients. *Arch. Med. Sci. AMS* 9 398–403. 10.5114/aoms.2012.28658 23847658 PMC3701961

[B54] Salmela-Aro K. Kiuru N. Leskinen E. Nurmi J. E. (2009). School burnout inventory (SBI) reliability and validity. *Eur. J. Psychol. Assess.* 25 48–57. 10.1027/1015-5759.25.1.48

[B55] Samejima F. (1969). Estimation of latent ability using a response pattern of graded scores. *Psychometrika Monogr. Suppl.* 34 1–97. 10.1007/bf03372160

[B56] Savic I. (2015). Structural changes of the brain in relation to occupational stress. *Cereb. Cortex* 25 1554–1564. 10.1093/cercor/bht348 24352030

[B57] Schaufeli W. B. Leiter M. P. Maslach C. (2009). Burnout: 35 years of research and practice. *Career Dev. Int.* 14 204–220. 10.1108/13620430910966406

[B58] Schaufeli W. B. Leiter M. P. Maslach C. Jackson S. E. (1996). “MBI general survey,” in *Maslach Burnout Inventory Manual*, 3rd Edn, eds Maslach C. Jackson S. E. Leiter M. P. (Palo Alto, CA: Consulting Psychologists Press).

[B59] Schaufeli W. B. Martinez M. I. Pinto A. M. Salanova M. Bakker A. B. (2002). Burnout and engagement in university students – A cross national study. *J. Cross-Cult. Psychol.* 33 464–481. 10.1177/0022022102033005003

[B60] Schaufeli W. B. Taris T. W. (2005). The conceptualization and measurement of burnout: common ground and worlds apart. *Work & Stress* 19 256–262. 10.1080/02678370500385913

[B61] Schwenk T. L. Davis L. Wimsatt L. A. (2010). Depression, stigma, and suicidal ideation in medical students. *JAMA* 304 1181–1190. 20841531 10.1001/jama.2010.1300

[B62] Toker S. Melamed S. Berliner S. Zeltser D. Shapira I. (2012). Burnout and risk of coronary heart disease: a prospective study of 8838 employees. *Psychos. Med.* 74 840–847. 10.1097/PSY.0b013e31826c3174 23006431

[B63] Tucker J. R. Hammer J. H. Vogel D. Bitman R. Wade N. G. Maier E. (2013). Disentangling self-stigma: are mental illness and help-seeking self-stigmas different? *J. Counsel. Psychol.* 60 520–531. 10.1037/a0033555 23815629

[B64] U. S. Department of Education, National Center for Education Statistics (2017). *State Nonfiscal Survey of Public Elementary and Secondary Education, 1990–91 Through 2014–2015.* Washington, DC: U. S. Department of Education, National Center for Education Statistics.

[B65] Walburg V. (2014). Burnout among high school students: a literature review. *Children Youth Serv. Rev.* 42 28–33. 10.1016/j.childyouth.2014.03.020

[B66] World Health Organization (2019). *Burn-Out an “Occupational Phenomenon”: International Classification of Diseases.* Geneva: World Health Organization Available at: https://www.who.int/mental_health/evidence/burn-out/en/

